# Improving Oral Hygiene Skills by Computer-Based Training: A Randomized Controlled Comparison of the Modified Bass and the Fones Techniques

**DOI:** 10.1371/journal.pone.0037072

**Published:** 2012-05-21

**Authors:** Daniela Harnacke, Simona Mitter, Marc Lehner, Jörn Munzert, Renate Deinzer

**Affiliations:** 1 Institute of Medical Psychology, University of Giessen, Giessen, Germany; 2 Institute of Sports Sciences, University of Giessen, Giessen, Germany; University of Southern California, United States of America

## Abstract

**Background:**

Gingivitis and other plaque-associated diseases have a high prevalence in western communities even though the majority of adults report daily oral hygiene. This indicates a lack of oral hygiene skills. Currently, there is no clear evidence as to which brushing technique would bring about the best oral hygiene skills. While the modified Bass technique is often recommended by dentists and in textbooks, the Fones technique is often recommended in patient brochures. Still, standardized comparisons of the effectiveness of teaching these techniques are lacking.

**Methodology/Principal Findings:**

In a final sample of n = 56 students, this multidisciplinary, randomized, examiner-blinded, controlled study compared the effects of parallel and standardized interactive computer presentations teaching either the Fones or the modified Bass technique. A control group was taught the basics of tooth brushing alone. Oral hygiene skills (remaining plaque after thorough oral hygiene) and gingivitis were assessed at baseline and 6, 12, and 28 weeks after the intervention. We found a significant group×time interaction for gingivitis (F(4/102) = 3.267; p = 0.016; ε = 0.957; η^2^ = 0.114) and a significant main effect of group for oral hygiene skills (F(2/51) = 7.088; p = 0.002; η^2^ = 0.218). Fones was superior to Bass; Bass did not differ from the control group. Group differences were most prominent after 6 and 12 weeks.

**Conclusions/Significance:**

The present trial indicates an advantage of teaching the Fones as compared to the modified Bass technique with respect to oral hygiene skills and gingivitis. Future studies are needed to analyze whether the disadvantage of teaching the Bass technique observed here is restricted to the teaching method employed.

**Trial Registration:**

German Clinical Trials Register DRKS00003488

## Introduction

Though daily plaque removal is considered to be important for oral health [1], [2], representative studies indicate this goal is not achieved by most patients. Approximately 90% of German adults suffer from gingivitis and 30%–70% from periodontitis [3]. Other countries report similar figures [4]. Contemporaneously, 70% of German patients report brushing their teeth twice a day [5] indicating that most patients do not sufficiently remove all plaque deposits. Recent studies by our group support the notion that skill deficits may play an important role here. When we asked a representative German sample whether they knew any brushing technique, more than 30% responded negatively [6]. Students were found to brush their teeth rather unsystematically in a video observation study [7] and to remove no more than 60% of marginal plaque deposits when asked to brush to the best of their abilities [8]. Current data thus suggest deficits in oral hygiene skills which might be overcome by teaching brushing techniques.

Today, there is little evidence as to which brushing technique would bring about the best results for oral hygiene at home, e.g. [9], [10]. The few studies directly comparing brushing techniques suffer from methodological shortcomings like lack of control groups [11], non-blinded examiners [12], [13] or confounders and missing standardization [14]. We thus decided to compare the effects of computer-based training. A major advantage of computer-based training is its high degree of standardization, its repeatability, and its transparency. This is what has been called for in research into oral hygiene techniques for a long time, e.g. [15]. We compared training in the modified Bass technique and the Fones technique. The Fones technique seems to be the one best known to German adults [6] and is also considered a standard technique, e.g. [16]. The modified Bass technique, on the other hand, is often recommended as being particularly efficient in removing plaque at the gingival margin and thereby in preventing periodontal lesions, e.g. [16], [17].

According to arguments proposed, for example, by Renz et al. [18], we integrated psychological knowledge into the design of the interventions and extended their appeal by also including an expert in motor control and movement learning.

Teaching brushing techniques is a complex and time-consuming procedure. From the perspective of movement sciences, skill training makes many repetitions of the same movements necessary in order to incorporate them into the motor program. This high number of repetitions is seen to be a prerequisite for automation of skills [19]. In face-to-face training it is difficult to motivate both the trainer and the trainee to repeat the same movement again and again and to practice the movements in very small steps, which would be desirable from a movement sciences perspective. This issue has been widely discussed as part versus whole practice [20]. Computer-based training might help to overcome this disadvantage and some others as well. While having only the computer as a training device, the trainee may choose his or her own learning tempo to practice the movements. Furthermore, the trainee does not feel observed when performing the movements in front of a computer. Thus, adverse effects of the social interaction, like feelings of embarrassment, can be diminished, thereby allowing the trainee to fully concentrate on the training and not on the consequences of the social interaction.

We hypothesized that both computer-based training of the modified Bass technique and the Fones technique would improve oral hygiene skills and gingival health as compared to controls. Furthermore, we aimed to investigate whether there would be a difference in the effectiveness of computer-based training of Bass vs. Fones. To find out how long training effects persist without any further intervention, we assessed skills and gingival health 6, 12, and 28 weeks after training.

## Materials and Methods

The protocol for this trial and supporting Consort checklist are available as supporting information; see Checklist S1 and Protocol S1.

### Participants

N = 67 students at the University of Giessen provided informed written consent and fulfilled the following inclusion and exclusion criteria: at least 20 of their own teeth, 10 or more teeth showing plaque or bleeding, no study of dentistry, no smoking, no electrical tooth brushing, no dental treatment affecting gingival health or oral hygiene throughout the study (participant flow is shown as supporting information in CONSORT Flow Diagram S1). Participants were promised a monetary compensation (€ 50) in order to cover for the investments of time and travel costs. They were also promised a small gift of oral hygiene products as appreciation for their help in the study. Participants were recruited with the help of postings on the campus and announcements in local magazines. In these postings and announcements some information (getting a professional tooth cleaning; examination points; monetary compensation and gift of oral hygiene products) and inclusion criteria were already given (number of teeth, no smoking and to be a student). Students who responded to the postings and announcements received additional information and were asked about inclusion criteria (except of plaque and bleeding). Participants who met the inclusion criteria were invited to a first appointment. The study took place in laboratories of the Institute of Medical Psychology, University of Giessen, Germany.

The study protocol was conducted according to the principles of the Declaration of Helsinki and approved by the local Ethics Committee of the medical department of the University of Giessen (91/09). All participants provided informed written consent.

### Independent Variable

Participants were randomly assigned to a PowerPoint-based training of either Fones technique, modified Bass technique, or basics of tooth brushing alone (control); groups were stratified with respect to oral hygiene skills at baseline (for assessment see below) and gender. Tickets for randomization were put in identical, opaque boxes and were drawn by a person not involved in the study.

To reduce participant expectations (and confounders associated with them), participants were not informed about the three conditions or the hypotheses to be tested. Furthermore, they were not told the common name of the technique taught, to prevent them obtaining further information via the Internet. All participants received the same brand of toothbrush (Elmex InterX, GABA, Germany), toothpaste (Elmex, GABA, Germany), and dental floss (Elmex waxed and unwaxed, GABA, Germany) for oral hygiene at home and were asked not to use additional aids like mouth rinsing solution, etc.

Participants can navigate the PowerPoint based training back and forth and repeat every part as often as they want to. The presentations comprise written text, oral explanations, pictures, and videos. For skill acquisition, multimodal training devices have proven to be remarkably efficient [21]. As brushing is demonstrated in videos and photographs, separate presentations for left handers and right handers are provided (pictures in left handers' presentations are mirror images of the original pictures taken of right handers). A mirror is provided to allow for exercising with visual control of what is seen in the presentation. Participants are asked at several points to exercise immediately what they see (see Presentations S1). At the end of the presentation, participants are asked to apply the technique from now on whenever brushing their teeth. When they have finished the training, participants receive a brochure to be able look up major aspects of their presentations at home.

The content of the three training programs is provided in detail as supporting information (see Presentations S1). Every training program starts with twelve slides explaining the structure of the presentation and some basics of tooth brushing (called 1×1 of tooth brushing), namely sites to be cleaned, devices that can reach them, systematics of tooth brushing and their advantages, and brushing pressure. In the control condition, the training ends after these slides. In the Fones and Bass conditions, the training is continued with a further 25 slides. Presentations for the modified Bass technique and the Fones technique are parallel in all major aspects (i.e., number of slides (25) and videos (7), design of slides, persons demonstrating the technique, time of repetitions, words to encourage participants to try the technique) beside the technique shown.

### Dependent Variables

Dependent variables were assessed by calibrated examiners blind to the condition of the participants.

**Figure 1:**
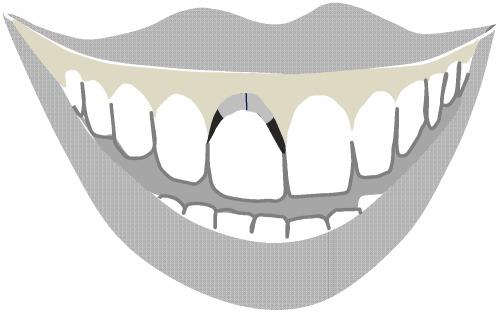
Assessment of the Marginal Plaque Index (Deinzer et al., submitted). The gingival margin is divided into four equal sections. For each section, the presence or absence of disclosed plaque is registered. Eight sections per tooth are registered: vestibular cervical (grey): two sections; vestibular approximal (black): two sections; oral cervical: two sections; oral approximal: two sections.

As an indicator of gingivitis, the papillary bleeding index (PBI) by Saxer & Mühlemann [22], modified by Rateitschak [23], was assessed at all sites. To assess oral hygiene skills, participants were asked to clean their teeth as thoroughly as possible. They were provided with several devices like a tooth brush, tooth paste, and dental floss and were allowed to use their own devices. Afterwards, remaining plaque was disclosed by Mira-2-Ton ®-solution (Hager & Werken, GMBH & Co, Duisburg, Germany) and staining was assessed by the Turesky [24] modification of the plaque index (TQHI) of Quigley & Hein [25] and the marginal plaque index (MPI; Deinzer et al., submitted) which assesses the presences or absence of staining at the gingival margin and provides good validity coefficients (see Figure 1).

### Design and procedure

This was a randomized, stratified (skills and gender), examiner-blinded, controlled study conducted in Germany. At baseline, participants were examined for eligibility (see above) and provided written informed consent. The dependent variables were first assessed. Afterwards, all participants were shown how to use dental floss by means of a video (provided by GABA international) and were checked and corrected by a dentist (S.M.) and asked to floss all teeth daily from now on. The next appointment comprised a professional tooth cleaning (removal of plaque and supragingival calculus and polishing) and random allocated to one of the interventions, which were launched by a person not further involved in the study. To maintain examiner blindness, participants were asked not to communicate the content of the presentation to anyone, especially not to their examiner and not to ask the examiner any questions regarding the presentation. Compliance with this instruction was excellent. Dependent variables were assessed 6, 12, and 28 weeks after this visit. At the end of the study, after they had received their monetary compensation, participants were asked to answer a short questionnaire where they should describe the technique they learned, the degree of their adherence (“I applied the technique consistently/inconsistently”) and reasons why they did or did not adhere.

### Statistical Analyses

The unit of analysis is the person. Analyses respectively refer to percentage of sites showing gingivitis (positive bleeding response) measured by PBI (papillary bleeding index), percentage of sites showing staining as assessed by the MPI, and mean score of the TQHI. The mean TQHI is computed irrespective of the ordinal scaling of this measure to provide better international comparability (in most international publications the mean TQHI is reported). Furthermore, this measure correlates well with other interval scaled plaque measures [26], [27] Because of the more detailed assessment of plaque deposits at the gingival margin, the MPI was taken as the primary and the TQHI as the secondary dependent variable to test for treatment effects on oral hygiene skills. Significance was considered with p≤0.05 and tentative significance with p≤0.10. Group size was determined to allow for the detection of large effect sizes (f≥0.40) with an α-error probability of 5% and a test-power of 80%.

Statistical analyses were run with SPSS 17.0 (SPSS Inc.). All parameters were tested for normal distribution by the Kolmogorov-Smirnov Test and were found not to deviate from the normal distribution assumption (all p>0.05). To examine baseline differences, ANOVAs and Chi^2^ Tests were run as indicated by the variable characteristics. Respective baseline values were included as covariates in all analyses. To analyse overall intervention effects, two factorial (group×time) analyses of covariance (ANCOVA) were run and corrected for non-sphericity by applying Greenhouse-Geisser's ε. In the case of significant results of overall analyses, one-factorial ANCOVAs and pairwise comparisons were computed to assess how long group differences persist without further intervention and which groups differed. Partial η^2^ is reported as measure of effect size.

## Results

Table 1 provides baseline characteristics of participants. Groups did not differ statistically significant in any of the variables (all p>0.262).

**Table 1:** Group differences at baseline.

Groups Variables	Control (n = 19)	Fones (n = 19)	Bass (n = 18)
Age*	23.53 (2.39)	23.21 (1.75)	22.94 (2.16)
Gender (male/female)	3m, 16f	4m, 15f	5m, 13f
PBI, bleeding sites*	19.73% (9.58)	22.04% (8.80)	24.80% (9.62)
MPI, sections with staining*	68.53% (14.18)	71.30% (13.70)	68.62% (12.78)
TQHI, mean score*	2.57 (0.56)	2.52 (0.43)	2.50 (0.50)

TQHI: Turesky modification of the Quigley&Hein Index; MPI: Marginal plaque Index; PBI: Papillary bleeding index;

* mean (standard deviation).

### Overall intervention effects

**Figure 2:**
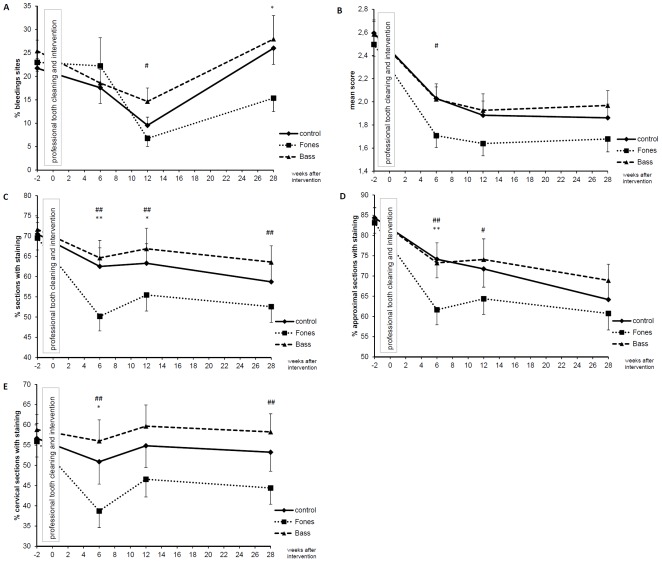
Papillary Bleeding index (PBI;A), oral hygiene skills measured by TQHI (B), MPI all sections (C), MPI approximal sections (D), and MPI cervical sections (E) over time. Mean and standard error of the mean of percentage of sites with bleeding (PBI>0), mean score of the Turesky modification of the Quigley & Hein Index (TQHI) and of percentage of sections showing staining as assessed by the MPI are shown for all groups (control n = 19; Fones n = 19; Bass n = 18) at baseline, 6, 12, and 28 weeks after intervention. Pairwise ANCOVAs are coded as following: *^,^**p≤0.05, p≤0.01 Fones vs. control; ^#,##^p≤0.05, p≤0.01 Fones vs. Bass; ^+,++^p≤0.05, p≤0.01 Bass vs. control.

Overall ANCOVAs revealed a significant group×time interaction (F(4/102) = 3.267; p = 0.016; ε = 0.957; η^2^ = 0.114) for gingivitis (Figure 2) and a significant main effect of group for oral hygiene skills measured with the MPI (F(2/51) = 7.088; p = 0.002; η^2^ = 0.218). No significant effect was observed for hygiene skills measured with the TQHI (F(2/51) = 2.204; p = 0.121; η^2^ = 0.080) (see Figure 2). Separate analyses for approximal and cervical sections of the gingival margin, as assessed by the MPI, revealed significant main effects of group for both approximal (F(2/51) = 4.435; p = 0.017; η^2^ = 0.148) and cervical sections (F(2/51) = 7.776; p = 0.001; η^2^ = 0.234).

### Effects after 6, 12, and 28 weeks

Table 2 presents results of univariate ANCOVAs for each point in time. In these analyses, significant group differences were observed for the PBI after 28 weeks. The MPI revealed significant group differences for all sections together and for cervical sections alone after 6, 12, and 28 weeks. For approximal sections, significant group differences were observed after 6 and 12 weeks. Results of pairwise group comparisons are given in Figure 2. At no time did Bass differ significantly from control. Fones differed significantly from control with respect to PBI after 28 weeks and with respect to skills after 6 and 12 weeks. Significant differences between Bass and Fones were observed with respect to gingival health after 12 weeks and with respect to skills after 6, 12, and 28 weeks. From these analyses, training of Fones turned out to be superior to training of basics of tooth brushing alone (control) and training of Bass.

**Table 2:** Results of ANCOVAs comparing groups at each point in time.

	F-Statistics	η^2^	P
**Gingivitis (% sites with bleeding)**			
Papillary bleeding index			
6 weeks	F(2/52) = 0.539	0.020	0.587
12 weeks	F(2/52) = 2.829	0.098	0.068
28 weeks	F(2/52) = 3.582	0.121	0.035*
**Oral hygiene skills**			
TQHI (mean score)			
6 weeks	F(2/52) = 3.124	0.107	0.052#
12 weeks	F(2/52) = 1.801	0.065	0.175#
28 weeks	F(2/52) = 1.763	0.064	0.182#
MPI (% staining) - all sections			
6 weeks	F(2/52) = 7.323	0.220	0.002*
12 weeks	F(2/52) = 4.808	0.156	0.012*
28 weeks	F(2/52) = 3.991	0.133	0.024*
- approximal sections			
6 weeks	F(2/52) = 7.016	0.213	0.002*
12 weeks	F(2/52) = 3.172	0.109	0.050*
28 weeks	F(2/52) = 2.058	0.073	0.138
- cervical sections			
6 weeks	F(2/52) = 5.736	0.181	0.006*
12 weeks	F(2/52) = 5.313	0.170	0.008*
28 weeks	F(2/52) = 5.043	0.162	0.010*

TQHI: Turesky modification of the Quigley&Hein Index; MPI: Marginal plaque Index;

* significant differences;

# an ANCOVA including all points in time reveals no significant result (see Text), comparisons for each point in time are thereby presented for exploratory reasons, only.

### Self-reported adherence

Both in the control and in the Fones group, five participants reported not having applied the technique consistently, while within the Bass group eleven persons reported non-adherence. In all groups, the reasons reported most often as main reason for non-adherence are related to the subjective expenses of the technique (e.g., time pressure, examination stress, idleness; control 5, Fones 5, Bass 8). Three persons of the Bass group reported unpleasant feelings (“unfriendly to the gingiva”) as the main reason for non-adherence.

## Discussion

This study aimed to compare the respective effects of three interactive computer presentations teaching the modified Bass or the Fones technique or the basics of oral hygiene alone.

Our hypothesis is confirmed only in part. Only the computer-based training of Fones turned out to be effective as compared to a control group but not the computer-based training of the modified Bass technique. Aiming to show whether computer-based training of Fones and Bass would differ in their effectiveness, we found superior results for the training of the Fones technique. Regarding the duration of effects, training of Fones turned out to be superior to training of the modified Bass technique throughout the experiment. Considering skills, maximum differences between Fones and control groups were observed after 6 weeks, while regarding gingival health differences, reached a maximum after 28 weeks.

Our result is surprising with respect to the low effectiveness of teaching the modified Bass technique. Even though participants in the Bass group received an add-on compared to what the control group had been taught, their results were in no way superior. Thus, it seems as if teaching the Bass technique is of no advantage over teaching the basics of tooth brushing alone. Instead, these groups return to baseline values of bleeding at the end of the study. In contrast, teaching the Fones technique brought about a clear advantage in terms of gingivitis and hygiene skills. This result is remarkable and warrants closer inspection.

Our findings are in line with those of Arai & Kinoshita [13], who compared remaining plaque after brushing with the Bass and the Fones techniques and found the Fones technique to be superior. It is interesting that their participants were dental students and dental staff, persons who should know all techniques pretty well. Some other studies are less conclusive, as they lack control groups and standardized instructions. Furthermore, gingivitis as an indicator of habitual oral hygiene is not assessed in these studies nor are oral hygiene skills as a premise of successful hygiene [11], [12], [28], [29].

There are several possibilities as to why Fones turns out to be superior in the present study. First and most importantly, the Fones technique is the one best known in Germany [6]. Thus, teaching Fones might have been a repetition and reminder to our participants, while teaching Bass might have meant to them a completely new way of brushing their teeth. However, while this may explain superiority of the Fones technique in our study, it does not explain the lack of effects of Bass against the control. Perhaps this technique is more difficult to integrate into everyday life. This has been indicated by Arai & Kinoshita [13] and indeed in the present study more participants in the Bass than in the other groups reported non-adherence. In the present study, the respective technique was taught only once and the participants were not checked by another person. Instead, participants themselves checked in the mirror provided beneath the computer whether they were performing the technique as shown in the computer presentation. Perhaps for a technique as difficult as the Bass technique, checking by a dentist would be necessary. Furthermore, even though participants were provided with a brochure to be able to call to mind the most important features of the technique at home, this might have been too little effort to teach a completely new technique. Future studies should find out whether teaching the Bass technique would bring about advantages if more than one session were applied, if a dentist checked what they had learned and if patients were encouraged on a more regular basis to adopt this technique.

The suggested re-encouragement might have been useful in the Fones group, too. After an initial improvement of hygiene skills, no further improvement is seen throughout the rest of the study. Future studies are needed to further elucidate this finding and to determine which interventions would be effective in further enhancing hygiene skills.

One limitation of our study is that the Fones technique seems to be better known in adults. It therefore remains open whether teaching Bass would have brought about better results if this technique had also been already known. Still, for dental practice it is important to realize that teaching Fones seems to fall on prepared ground, while teaching Bass for most patients means entering virgin soil and may thus require much more investment at the beginning. One should realize, however, that computer-based training as provided in this study took about 45 minutes, which is already quite an investment, at least for the patient, and exceeds by far what is commonly provided in dental practice for oral hygiene skills training in face-to-face settings. Another limitation is our study population, which was restricted to students, thereby challenging the external validity of our study. Additionally, much more women than men volunteered for this study which can only partially attributed to an uneven distribution of men and women (1∶2) within students of our university. Future studies should include more men, participants of different ages, education, and familiarity with a computer to demonstrate whether positive effects of the Fones training can be observed in these groups, too. With respect to the lack of effectiveness of the Bass technique, there is no reason to expect that other populations would show more advantageous results. Instead, the group we analyzed is expected to show at least average if not above average cognitive and motor learning capacities, attributes promoting rather than hindering learning of a complicated motor skill [20]. A further limitation of our study lies in the way of teaching itself. Our computer presentation provides detailed instructions, which allows for several repetitions and for adoption at the individual learning rate in that the participant is able to navigate at his or her own tempo. Indeed, it has been shown that self-monitored practice schedules may enhance skill acquisition [30], [31]. Furthermore, especially when learning a new skill, a high degree of detail is desirable and it is difficult to provide this detail in a face-to-face interaction. We thus decided on a computer presentation. However, in doing so we waived the effect of a direct patient-physician interaction, which might have helped to improve compliance, e.g. [32]. Similarly we waived any further measures to improve oral hygiene compliance, like teaching advantages of sufficient oral hygiene or working out implementation intentions, as suggested by e.g. Gollwitzer [33], or self-regulation and motivational interviewing, as recently demonstrated by Godard et al. [34]. We also did not employ measures to re-motivate participants, like oral hygiene feedback or repeated teaching sessions. Employing all of these measures might have improved our study results and led to better results with respect to skills and gingivitis. Such an improvement would be mandatory as even in the best (i.e. Fones) group participants never reached better values than a mean of 50% of marginal sections cleaned. Even though this is a considerable improvement on baseline, and even though gingivitis rates remain pretty low in this group, there is still plenty of room for further improvement.

Irrespective of these limitations, the study provides some important insights. First of all, we demonstrated that a computer presentation teaching the Fones technique brought about significant improvements in skills and gingivitis in a population of students. Secondly, we found teaching the modified Bass technique in a parallel manner to be of no advantage over teaching oral hygiene basics alone. Thus, the results of the present randomized, controlled trial do not encourage teaching the modified Bass technique, at least via a computer presentation. Future studies are needed to analyze whether this disadvantage of Bass is restricted to the teaching method employed here. Furthermore, our results raise several other questions to be answered in future studies, like the effects of the same teaching methods in different populations, the effects of additional measures to improve oral hygiene (motivation techniques etc.), and the different efforts one should make when teaching a well-known technique like Fones and a less known technique like Bass.

## Supporting Information

Checklist S1 **CONSORT Checklist.**
(DOC)

CONSORT Flow Diagram S1 **CONSORT Flow of Participants.**
(DOC)

Presentations S1 **Content of the slides in the PowerPoint based trainings for oral hygiene.**
(DOC)

Protocol S1 **Study Protocol.**
(DOC)
