# Supplementary material for: Improving Oral Hygiene Skills by Computer-Based Training: A Randomized Controlled Comparison of the Modified Bass and the Fones Techniques
Source: PLoS One. 2012 May 21;7(5):e37072. doi: 10.1371/journal.pone.0037072 (PMC3357431; doi:10.1371/journal.pone.0037072)
Supplement: CONSORT Flow Diagram S1 — CONSORT Flow of Participants. (DOC) [file pone.0037072.s002.doc]

**Enrollment:170**

**Fones**

**Control**

**mod. Bass**

Assessed for eligibility (n=133)

Excluded (n=66)

♦  Not meeting inclusion criteria (n=14)

♦  Declined to participate (n=21)

♦  Other reasons (n=31)

Analysed (n=19)

Allocated to intervention (n=23)

♦ Received allocated intervention (n=23)

Allocated to intervention (n=22)

♦ Received allocated intervention (n=21)

♦ Did not receive allocated intervention (participant not available) (n=1)

Analysed (n=19)

Randomized (n= 67)

Allocated to intervention (n=22)

♦ Received allocated intervention (n=20)

♦ Did not receive allocated intervention (participants not available) (n=2)

**Follow-Up after 6 weeks**

Lost to follow-up (participant not available) (n=4)

Lost to follow-up (participant not available) (n=1)

Lost to follow-up (participant not available) (n=2)

Lost to follow-up (Participant not able to keep appointment, continues study) (n=1)

**Follow-Up after 12 weeks**

Lost to follow-up (Participant reported gingival recession) (n=1)

Lost to follow-up (n=0)

Lost to follow-up (n=0)

**Follow-Up after 28 weeks**

Lost to follow-up (n=0)

Lost to follow-up (n=0)

Lost to follow-up (n=0)

**Analysis**

Analysed (n=18)
