# Supplementary material for: Improving Oral Hygiene Skills by Computer-Based Training: A Randomized Controlled Comparison of the Modified Bass and the Fones Techniques
Source: PLoS One. 2012 May 21;7(5):e37072. doi: 10.1371/journal.pone.0037072 (PMC3357431; doi:10.1371/journal.pone.0037072)
Supplement: Presentations S1 — Content of the slides in the PowerPoint based trainings for oral hygiene. (DOC) [file pone.0037072.s003.doc]

| *S4: Content of the slides in the PowerPoint based trainings for oral hygiene* | |
| --- | --- |
| **Slides No.** | **All presentations** |
| **1** | Title and short instruction on how to continue presentation |
| **2** | Overview |
| **3** | Instruction and explanation of structure of the presentation |
| **4** | Title of the presentation (1x1 of tooth brushing) |
| **5** | Outline of the presentation (Five surfaces, systematics and pressure) |
| **6** | Five surfaces of a tooth to be cleaned and where they are |
| **7** | … and how to reach (approximal via dental floss, other surfaces via dental brush) |
| **8** | Systematics of tooth brushing: find the last tooth of one jaw and start with it; develop a fixed sequence of brushing all surfaces |
| **9** | Recommended pressure for brushing: 200g; to be tested with a scale laced next to computer |
| **10** | Summary of the presentation (Five surfaces; systematics; pressure) |
| **Slides No.** | **Presentation teaching Basics of Tooth brushing only (control condition)** |
| **11** | Congratulation for what has been learned and reminder to practice it (considering systematics while brushing, brushing 2 times a day, use dental floss once a day); announcement that participant is going to receive a brochure summarizing what has been learned) |
| **12** | Acknowledgement for participation |

| **Slides No.** | **Presentation teaching Fones** | **Presentation teaching Bass** |
| --- | --- | --- |
| **11** | Title of the second part of the presentation (The Circular Technique – an effective way to clean your teeth) | Title of the second part of the presentation (The Jiggle Technique – an effective way to clean your teeth) |
| **12** | Outline of the presentation | Outline of the presentation |
| **13** | Introduction of the use of the mirror provided (controlling movements while repeating what is presented in the following) | Introduction of the use of the mirror provided (controlling movements while repeating what is presented in the following) |
| **14** | Explanation of the outline of each subsection: Introductory video to be watched, pictures explaining movements step by step, introduction to imitating what is seen whenever advised, closing video to be imitated while watching it | Explanation of the outline of each subsection: Introductory video to be watched, pictures explaining movements step by step, introduction to imitating what is seen whenever advised, closing video to be imitated while watching it |
| **15** | Introductory video (just to be watched) demonstrating cleaning of the vestibular surfaces (upper and lower jaw) | Introductory video (just to be watched) demonstrating cleaning of the vestibular surfaces (upper and lower jaw) |

| **16** | Explanation step by step for the left side: closing the alignment, finding the vestibular surface of the last tooth on the left side and putting the brush straight to the upper gingival margin | Explanation step by step for the left upper jaw: opening the mouth; finding the vestibular surface of the last tooth in the upper jaw left; placing the brush at an angle so as to feel the bristles reaching a little bit under the margin |
| --- | --- | --- |
| **17** | … painting circles on the teeth of the upper and lower jaw; taking care to touch the upper and lower margins; moving on tooth by tooth until the incisors are reached | … jiggling in small movements ten times back and forth on one tooth and then wiping out to the occlusal surfaces; repeating this on the same tooth; moving forward to the next tooth until the incisors are reached |
| **18** | Explanation step by step for the right side: brushing the same way on the right side and checking oneself in the mirror: circular movements; touching the margin; moving tooth by tooth | Explanation step by step for the right upper jaw: brushing the same way for the right upper jaw; finding the last tooth; placing the brush at an angle; jiggling and wiping out; repeating: placing the brush at an angle, jiggling and wiping out; tooth by tooth until incisors are reached |
| **19** | Video (to be imitated) demonstrating cleaning of the vestibular surfaces (upper and lower jaw) | Explaining step by step for the left lower jaw: finding the last tooth in the mirror; placing the brush at an angle, jiggling and wiping out; repeating: placing the brush at an angle, jiggling and wiping out; tooth by tooth until the incisors are reached |
| **20** | Introductory video (just to be watched) demonstrating cleaning of the oral surfaces | Explaining step by step for the right lower jaw: finding the last tooth; placing the brush at an angle, jiggling and wiping out; repeating, placing the brush at an angle, jiggling and wiping out; tooth by tooth until the incisors are reached |
| **21** | Explanation step by step for the left upper jaw: opening the mouth, finding the oral surface of the last tooth in the left upper jaw by looking in the mirror; putting the brush straight to the gingival margin | Video (to be imitated) demonstrating cleaning of the vestibular surfaces (upper and lower jaw) |
| **22** | …brushing with small circular movements; taking care to touch the upper and lower margin; moving on tooth by tooth until incisors are reached | Introductory video (just to be watched) demonstrating cleaning of the oral surfaces |
| **23** | Explaining step by step for the right upper jaw: brushing the same way on the right side; checking oneself in the mirror: finding the last tooth; touching the gingival margin; small circling movements; tooth by tooth until the incisors are reached | Explaining step by step for the left upper jaw oral surfaces: opening the mouth; finding the oral surface of the last tooth in the left upper jaw; placing the brush at an angle , jiggling and wiping out; repeating placing the brush at an angle, jiggling and wiping out; tooth by tooth until the incisors are reached |
| **24** | Explaining step by step for the left lower jaw: brushing the same way as the left upper jaw; checking oneself in the mirror: finding the last tooth; touching the gingival margin; small circular movements; tooth by tooth until the incisors are reached | Explaining step by step for the right upper jaw; brushing the same way the right upper jaw: finding the last tooth;, placing the brush at an angle, jiggling and wiping out; repeating: placing the brush at an angle , jiggling and wiping out; tooth by tooth until the incisors are reached |
| **25** | Explaining step by step for the right lower jaw: brushing the same way on the right site; checking oneself in the mirror: finding the last tooth; touching the gingival margin; small circular movements; tooth by tooth until the incisors are reached | Explaining step by step for the left lower jaw: finding the last tooth in the mirror; placing the brush at an angle, jiggling and wiping out; repeating: placing the brush at an, jiggling and wiping out; tooth by tooth until incisors are reached |
| **26** | Video (to be imitated) demonstrating cleaning of the oral surfaces (upper and lower jaw) | Explaining step by step for the right lower jaw: finding the last tooth; placing the brush at an angle, jiggling and wiping out; repeating: placing the brush at an angle, jiggling and wiping out; tooth by tooth until the incisors are reached |
| **27** | Introductory video (just to be watched) demonstrating cleaning of the occlusal surfaces | Video (to be imitated) demonstrating cleaning of the oral surfaces (upper and lower jaw) |
| **28** | Explanation step by step for the left upper jaw: open the mouth; finding the last tooth in the upper jaw left by looking in the mirror; brushing with scrubbing movements tooth by tooth until the incisors are reached | Introduction video (just to be watched) demonstrating cleaning of the occlusal surfaces |
| **29** | Explaining step by step for the right upper jaw: brushing the same way on the right side; checking oneself in the mirror: finding occlusal surface of the last tooth; scrubbing movements; tooth by tooth until the incisors are reached | Explaining step by step for the upper jaw: opening the mouth and looking in the mirror; finding the occlusal surface of the last tooth in the upper jaw; scrubbing the surfaces tooth by tooth until the incisors are reached; brushing the right upper jaw the same way |
| **30** | Explaining step by step for the left lower jaw: brushing the same way on lower; checking oneself in the mirror: finding occlusal surface of the last tooth; scrubbing movements; tooth by tooth until the incisors are reached; proceed in the same way in the right lower jaw | Explaining step by step for the lower jaw; brushing the same way the lower jaw: finding the last tooth; scrubbing the surfaces tooth by tooth until the incisors are reached; brushing the right lower jaw the same way |
| **31** | Video (to be imitated) demonstrating cleaning of the occlusal surfaces (upper and lower jaw) | Video (to be imitated) demonstrating cleaning of the occlusal surfaces (upper and lower jaw) |
| **32** | Summary (systematic; cleaning vestibular and oral surfaces with circular movements; scrubbing occlusal surfaces and always beginning at the last tooth) | Summary (systematic; vestibular and oral surface: placing the brush at an angle against the gum, moving back and forward and then wiping out; scrubbing occlusal surfaces and always beginning at the last tooth) |
| **33** | Final video (to be imitated) demonstrating cleaning of the vestibular, oral, and occlusal surfaces | Final video (to be imitated) demonstrating cleaning of the vestibular, oral, and occlusal surfaces |
| **34** | Congratulation for what has been learned and reminder to practice it (considering the technique while brushing, brushing 2 times a day, use dental floss once a day; announcement that participant is going to receive a brochure summarizing what has been learned) | Congratulation for what has been learned and reminder to practice it (considering the technique while brushing, brushing 2 times a day, use dental floss once a day; announcement that participant is going to receive a brochure summarizing what has been learned) |
| **35** | Acknowledgement for participation | Acknowledgement for participation |
