# Supplementary material for: Improving Oral Hygiene Skills by Computer-Based Training: A Randomized Controlled Comparison of the Modified Bass and the Fones Techniques
Source: PLoS One. 2012 May 21;7(5):e37072. doi: 10.1371/journal.pone.0037072 (PMC3357431; doi:10.1371/journal.pone.0037072)
Supplement: Protocol S1 — Study Protocol. (DOC) [file pone.0037072.s004.doc]

**Comparison of brushing techniques- a multidisciplinary, randomised study teaching oral hygiene skills**

**Trial Protocol**

**(Translation of the original German protocol plus list of amendments)**

**Aim of the Study**

The aim of the study is a longitudinal controlled comparison of effectiveness of different brushing techniques. Three of the best known brushing techniques (Stillman, Fones and modified Bass) should be compared with each other and a control group. When planning such a study one has to take into account, that you need more than only dental experts to teach a brushing technique. Furthermore it is necessary to implement knowledge of movement sciences such as best teaching of fine motor motion sequence. In addition it requires psychological expertise to teach information in a way that it can be optimal processed, remembered and transformed and motivational aspects to support the application in everyday life. The present study will follow this approach to connect these three disciplines. A PowerPoint presentation will be worked out for every technique (Stillman, Fones and Bass) taking into account in all presentations knowledge of dentistry, movement sciences and psychology. The presentations are constructed in a way that participants are able to get through the presentation by themselves and to follow them in his/her own tempo. Additionally, they are able to follow single training steps in a mirror placed nearby. Another aim of this study is to evaluate the success of teaching these techniques with the help of clinical parameters in the following period. An equal focus is placed on oral hygiene skills, actual oral hygiene and oral health of the participants.

**State of research**

Oral hygiene at home has a special role in prevention of plaque associated diseases, like for example gingivitis, periodontitis and tooth decay. Especially gingivitis and periodontitis receive particular significance, because their prevalence constantly increases in the last years (Micheelis et al., 2008). Latest representative survey shows that more than 90 % of adults and seniors have gingivitis. The prevalence of periodontitis differed between 30-70% depending on method of collecting data, analysed severity code und age group that was examined. This is quite surprising taking into account that daily plaque removal is deemed to be an effective method of prevention. At the same time 60 % of seniors and over 70% of adults state to practise oral hygiene twice a day (Micheelis & Schiffner, 2006). Current data of our working group show that the percentage of people who practice at least once a day oral hygiene is even higher (Deinzer et al., in press). If at least 70 % stated to practice daily oral hygiene and the same time the prevalence of disease associated with plaque is 90 % this indicates a lack of oral hygiene skills. The data available suggest that patients have not the necessary skills to remove all dental plaque deposits with their oral hygiene at home. Studies of our working group point into this direction, too. Students are not able to remove more than 60 % of marginal plaque deposits on average (Granrath, 2007). Even less favourable data have been found for patients who participate in a study of oral hygiene skills teaching in prophylaxis-oriented dental practices (Deinzer et al., in preparation).

The increasing prevalence of periodontal diseases despite of high brushing frequency can be attributed to a lack of oral hygiene skills. Regarding dental literature to get evidence for the most effective plaque reducing brushing technique, one will not find any evidence. Currently, there is no clear evidence as to which brushing technique would bring about the best oral hygiene skills (Staehle et al., 2007). Randomised controlled studies that compare the different brushing techniques are missing. The aim of the present study is to catch up on this lack of evidence.

**Eligibility criteria**

Participants should be students (except of dentistry), non-smokers, more than 20 own teeth, bleeding and/or plaque on more than 10 sites. Participants should be recruited via postings on the campus and announcements in local magazines.

**Study Design**

After inclusion participants receive in group meetings an instruction in the application of floss to reach a standardized interdental hygiene as far as possible. These skills will be checked several times within the next two weeks. Afterwards all participants receive a professional tooth cleaning and the instruction of one of the three tooth brushing techniques. Dependent variables will be assessed two, six, twelve and twenty-eight weeks after this intervention. All participants receive standardized tooth brushes, toothpaste and floss for the investigation period and they will be encouraged to use these for their daily oral hygiene. After fourteen weeks the materials will be changed and all participants receive new materials. Therefore the participants bring back their old materials.

**Variables that will be observed and determined, respectively**

**Independent variables**

Participants will be stratified by gender and handedness and randomly assigned to one of three groups (Stilman, Fones or Bass).

**Dependent variables**

Dependent variables are oral hygiene skills, actual oral hygiene and oral health. Turesky modification (1970) of plaque index of Quigley and Hein (1962) (TQHI) will be assessed to measure oral hygiene skills and actual oral hygiene. Dental plaque will be stained with Mira-2-Ton solution and to evaluate actual oral hygiene only dark blue stained sites will be evaluate. To measure oral hygiene skills participants will be requested to brush their teeth to the best of their abilities and afterwards the teeth will be stained with Mira-2-Ton solution again and now all staining will be assessed. As an indicator of oral health bleeding will be assessed with the papillary bleeding index by Saxer & Mühlemann modified by Rateitschak (1989).

**Control variables**

All dependent variables will be assessed at baseline. Additionally, periodontitis-related knowledge, dental self-efficacy, pros and cons, stages of change, decisional balance of oral hygiene (for these there are already validated measuring instrument of our working group (Deinzer et al., in preparation; Granrath, 2007; Deinzer et al., 2008)) will be assessed at baseline and 28 weeks after intervention.

**Primary outcome measure**

Primary outcome measure is oral health 28 weeks after intervention.

**Secondary outcome measure**

Secondary outcome measures are oral hygiene skills at 28 weeks and on the other appointments and oral health on the other appointments.

**Potential disturbance variables**

Oral hygiene skills, oral hygiene and oral health parameters could influence the outcome therefore they will be assessed at baseline. Periodontitis-related knowledge, dental self-efficacy, pros and cons, stages of change, decisional balance of oral hygiene will be evaluated with already validated measuring instrument of our working group (Deinzer et al., in preparation; Granrath, 2007; Deinzer et al., 2008) and controlled.

**Biometrical/Statistical Analyses**

Primary outcome measure is oral health 28 weeks after intervention. Secondary outcome measures are oral hygiene skills and oral hygiene at 28 weeks and on the other appointments and oral health on the other appointments. Percentage of unstained sites MPI and TQHI and percentage of sites without bleeding are assessed. The inference-statistical analyses shall be made by Prof. Dr. Renate Deinzer.

Statistical data analyses will be conveyed with analysis of variance. Additionally exploratory analyses follow to investigate the effect sizes of group differences.

**Sample size calculation**

Group size was determined to allow for the detection of large effect sizes with an α-error probability of p≤ 0.05 and a β-error probability of p≤ 0.20.

**Organisation**

- Study chairman and budgetary responsibility: Prof. Dr. Renate Deinzer
- Cooperating partners:
- Department of Conservative and Preventive Dentistry, Dental Clinic, Justus-Liebig-University, Giessen, Germany, Prof. Dr. Ganß
- Institute of Medical Psychology Justus-Liebig-University, Giessen, Germany, Prof. Dr. Renate Deinzer
- Institute of Sports Sciences, University of Giessen, Germany, Prof. Dr. Hans-Jörn Munzert

**Time schedule**

July 2009: preparation of training materials

August 2009 – May 2010: recruitment and examination of participants

June – July 2010: data analysis and research report

**Time flow:**

After inclusion participants receive in group meetings an instruction in the application of floss to reach a standardized interdental hygiene as far as possible. These skills will be checked several times within the next two weeks. Afterwards all participants receive a professional tooth cleaning and the instruction of one of the three tooth brushing techniques. Dependent variables will be assessed two, six, twelve and twenty-eight weeks after this intervention. All participants receive standardized tooth brushes, toothpaste and floss for the investigation period and they will be encouraged to use these for their daily oral hygiene. After fourteen weeks the materials will be changed and all participants receive new materials. Therefore the participants bring back their old materials.

**Privacy of study subjects**

Input of questionnaire data takes place in a pseudonymized form and they will be saved locked and separated from personal data.

**Participants safety**

Participants are not at risk in this study.

**Quality management**

All examination will be performed by calibrated examiners and a standardized evaluation of the validated questionnaires will be provided.

**Guidelines**

CONSORT-Guidelines.

**Publication**

An international publication of results is planned.

**Publication of the trial protocol**

A publication of the trial protocol and registering the study respectively is not planned yet.

**References**

Deinzer R, Micheelis W, Granrath N, Hoffmann T (2008) Knowledge of the German population on periodontal disease – Results of a representative survey. IDZ-Information 1: 2–27.

Granrath N (2007) Effects of different intervention strategies and modalities on psychological mediators and clinical indicators of oral health behavior. Results of three randomized intervention studies. [Auswirkungen verschiedener Interventionsstrategien und -modalitäten auf psychologische Mediatoren. Ergebnisse dreier randomisierter Interventionsstudien]. [Thesis] Düsseldorf: Heinrich-Heine-Universität. <http://docserv.uni-duesseldorf.de/servlets/DocumentServlet?id=3546>.

Micheelis, W., Hoffmann, T., Holtfreter, B., Kocher, T., Schroeder, E. (2008) Epiodemiological evaluation of periodontal load in Germany – attempt of balancing. [Zur epidemiologischen Einschätzung der Parodontitislast in Deutschland - Versuch einer Bilanzierung.] DZZ 63, 464-472.

Micheelis, W. & Schiffner, U. (2006): Vierte Deutsche Mundgesundheitsstudie. Köln: Deutscher Zahnärzteverlag..

Quigley GA, Hein JW (1962) Comparative cleansing efficiency of manual and power brushing. J Am Dent Assoc 65: 26–29.

Rateitschak KH (1989) Periodontology [Parodontologie]. Stuttgart:Thieme. 69-70 pp.

Saxer UP, Mühlemann HR (1975) Motivation und Aufklärung. Schweiz Monatsschr Zahnmed 85: 905–919.

Staehle HJ, Schiffner U, Dörfer CE (2007) Mechanical tooth care at home and oral hygiene. Statement of the German Society for Dental and Oral Medicine (DGZMK) [Häusliche mechanische Zahn- und Mundpflege. Stellungnahme der Deutschen Gesellschaft für Zahn-, Mund- und Kieferheilkunde (DGZMK)]. Deut Zahnaezrtl Z 62: 616–620.

Turesky S, Glickman I, Sandberg R (1972) In vitro chemical inhibition of plaque formation. J Periodontol 43: 263–269.

**List of Amendments**

1.) Presentations of the Stillman technique turned out to be very similar to those of the Bass technique. Indeed, pictures and videos of the two techniques were hardly to differentiate. We thus decided to omit the Stillman technique and to inculde a control group instead, which was taught only basics of tooth brushing and no specific brushing technique.

2.) Data on oral hygiene (dark blue staining prior to tooth brushing) were excluded from analyses due to a misunderstanding when assessing oral hygiene. While maufacturer proposes to assess all blue staining in order to assess older deposits we assessed dark blue staining, only. Since gingivitis is considered another good clinical indicator for oral hygiene we focused this parameter instead.

3.) Data of the two-week-assessment point were omitted from analyses as this point in time turned out to be too close to the professional tooth cleaning with nearly no bleeding and very low degree of staining after tooth brushing (indeed, the loweset observed in all groups throughout the study) both obviously reflecting the favorable after-effects of the professional tooth cleaning rather than oral hygiene activities of the participants.
